# Preface: causes of obesity, theories, conjectures and evidence

**DOI:** 10.1098/rstb.2022.0200

**Published:** 2023-09-11

**Authors:** David B. Allison, Thorkild I. A. Sørensen, Kevin D. Hall, John R. Speakman

**Affiliations:** ^1^ School of Public Health, Indiana University, Bloomington, IN, USA; ^2^ Department of Public Health, University of Copenhagen, Copenhagen, Denmark; ^3^ Novo Nordisk Foundation Center for Basic Metabolic Research, University of Copenhagen, Copenhagen, Denmark; ^4^ National Institute of Diabetes and Digestive and Kidney Diseases, National Institutes of Health, Bethesda, MD, USA; ^5^ Shenzhen Key Laboratory of Metabolic Health, Center for Energy Metabolism and Reproduction, Shenzhen Institutes of Advanced Technology, Chinese Academy of Sciences, Shenzhen, People's Republic of China; ^6^ School of Biological Sciences, University of Aberdeen, Aberdeen, UK; ^7^ State Key Laboratory of Molecular Developmental Biology, Institute of Genetics and Developmental Biology, Chinese Academy of Sciences, Beijing, People's Republic of China; ^8^ CAS Center of Excellence in Animal Evolution and Genetics, Kunming, People's Republic of China

As we open this dual volume on *Causes of obesity: theories, conjectures and evidence,* which consists of papers from the speakers at a Royal Society discussion meeting held in October 2022 (see https://royalsociety.org/science-events-and-lectures/2022/10/causes-obesity/), we thank the Royal Society for their extraordinary support. This meeting was described by some as the best that they had attended in years. Dr Van Hubbard, Rear Admiral (ret.), US Public Health Service, NIDDK participated and wrote ‘I thought I would send a quick note to let you know the meeting in London has been great. It is one of the best workshops I have attended.’ Two anonymous attendees wrote ‘This was a FANTASTIC meeting from start to finish! I attended virtually and it was the best virtual experience I've ever had. The topics were fascinating and I learned …’ and ‘…people with opposing views and strong opinions on matters I personally find highly unlikely were invited. I really appreciated that because our first job is to try to find consensus and challenge our ideas to find the answers and compromises that benefit the public.’ For us on this organizing committee, it was one of the highlights of our careers and we memorialize the thoughts with eagerness here.

Let us unpack what the conference was about and why we believe it was and is still so important and generated so much enthusiasm.

Unlike other diseases whose causes are generally appreciated to have complex etiologies, obesity seems more likely to be viewed as having simple or obvious causes. Often, obesity is attributed to gluttony and sloth, two of the canonical seven deadly sins, and thus historically cast as a result of moral failure of righteous willpower. The focus of much of the history of obesity science [1] on energy intake and expenditure has perhaps inadvertently fed into such beliefs. Although energy intake and expenditure are vitally important, scientists have increasingly come to appreciate that appeals to conscious control neither seem logically defensible nor commensurate with decades of accumulating data regarding long-term regulation of food intake or energy expenditure [2,3].

Unlike other diseases, obesity and its purported causes are closely related to personal anecdotal experiences with eating, nutrition, exercise, and body habitus. Thus, anecdotal experiences often replace expertise, evidence, or the product of deep study. Which of us would opine without careful study on the cause of atrial fibrillation, beta cell dysfunction, et cetera? and yet how many of us have observed others do this with respect to obesity, and perhaps we have occasionally done so ourselves. Let us take a deep breath and recommit to thinking about obesity in scientific terms. The same eschewing of untested assumptions, the same questioning of premises, the same removal of simply saying ‘it is obvious’ as if that constituted evidence, the same rejection of ipse dixit statements, the same demand for research rigor is warranted.

Let us unpack this title of this conference further. First, it is about causes. This alone can mean many different things to many people. In his poetic book, ‘From Darwin to Derrida’ Professor David Haig talks about the many different meanings we may have when we say, why does this occur, or what is the cause of this, or what is the causal effect of this on that? Do we mean evolutionary explanations such as how did we as a species get to be what we are? or do we mean mechanisms whereby we ask by what physical processes does something occur? or do we adopt the Rubin causal model [4] and mean counterfactuals, i.e. how would things have been different had it not been for this postulated causal factor and define that difference as the causal effect? There are yet other ways of conceiving of cause, but these are some of the most common. Notably, even with causes, we stayed focused on the causes of obesity *per se*, deliberately not emphasizing other important themes such as why obesity may be a health hazard, nor what to do about obesity, neither in prevention or treatment.

The second word to unpack is obesity. This was not a conference about nutrition in general, underweight, or modest variations in body habitus or energy stores. Although clearly many speakers including us touched on those issues. It was a conference about obesity *per se*, which implies a degree of excess body fat, but more than a degree of excess body fat. It also implies an excess of body fat where excess itself must be defined and is typically defined as an amount that causes deleterious health effects or reduced lifespan. Conference speakers chose to discuss obesity from two perspectives: at the level of the rising population prevalence of excess body fat as well as at the level of the individual acknowledging that there may be different subtypes of obesity with different etiologies. So, there is yet more to be defined, and there are causal factors implied in that definition as well.

The next word to unpack is theories. Many non-scientists use the word theory in the way scientists often use the word ‘model’, meaning an idea provided for discussion and testing that has some initial sense of plausibility, that cannot with current knowledge be ruled out. For discussion of the value of theories, see [5]. Others, including many mainstream scientists, when talking about things like the theory of relativity or the theory of natural selection from Darwin, recognize that these are far more robust conceptual frameworks that spin off many specific models and that while some specific models may or may not be in evidence, the overwhelming evidence indicates that these theories are, given our current knowledge, largely unshakable bulwarks.

That does not mean theories are not subject to refinement and revision, but true refutation is implausible given how well they are established. The laws of thermodynamics including and especially the first law, the law of energy conservation fit into that category. In that sense, they are underlying theoretical frameworks for much of what was discussed. In fact, often the theories presented and discussed at our conference dealt with evolutionary explanation of why we have obesity as a phenotype. Other things discussed are closer to the idea of theories in the sense that the general public often uses the word. We operated at both levels.

The next word to unpack is conjectures. Conjectures are vital in science. They move us forward. Ramanujan's Notebooks, Hilbert's 23 unsolved problems, Fermat's Last Theorem, all were conjectures. Even the Wright brothers implicit proposition that a flying machine of the type they conceived could be built and function was a conjecture. Jacob Bernoulli beautifully brought thinking about conjecture forward in his famous *Ars Conjectandi* [6]. Conjecture is vital [7]. We should applaud conjecture. It is often where the very best scientists have the very biggest impact.

As Asimov famously said (or is conjectured to have said) ‘The most exciting phrase to hear in science, the one that heralds new discoveries, is not ‘Eureka!’ (I found it!) but ‘That's funny …’ (see https://quoteinvestigator.com/2015/03/02/eureka-funny/). So, we gave free reign to conjecture. Yet for practical purposes, conjecture is good, but knowing is better. This is a phrase one of us (DBA) uses regularly and is the basis for a book his school distributes freely at this link [8]. So, we encouraged conjecture, but we also encouraged thinking how we can move from conjecture to knowing. What studies, what methods, what experiments would it take to move us toward greater knowledge, and most of all, we encourage distinguishing between conjecture and knowing.

The reader will find a rich diversity of opinions here. The reader will find theories in both senses of the word. The reader will find conjectures and facts pertinent to different ideas of causation. There will be no shortage of entertainment, of inspiring insights and of suggestions for key future studies. Enjoy the buffet!

## Data Availability

This article does not contain any additional data.
